# Angiogenesis, hereditary hemorrhagic telangiectasia and COVID-19

**DOI:** 10.1007/s10456-020-09755-5

**Published:** 2020-10-14

**Authors:** Antoni Riera-Mestre, Adriana Iriarte, Manuela Moreno, Raul del Castillo, Daniel López-Wolf

**Affiliations:** 1 Hereditary Hemorrhagic Telangiectasia Unit, Internal Medicine Department, Hospital Universitari de Bellvitge – IDIBELL, Feixa Llarga S/N, 08907 L’Hospitalet de Llobregat, Barcelona, Spain; 2 Faculty of Medicine and Health Sciences, Universitat de Barcelona, Barcelona, Spain; 3 RiHHTa (Computerized Registry of Hereditary Hemorrhagic Telangiectasia) Investigators. Rare Diseases Working Group of the Spanish Society of Internal Medicine, Madrid, Spain; 4 Internal Medicine Department, Hospital Clínico Universitario San Cecilio, Granada, Spain; 5 Otorhinolaryngology Department, Hospital Universitario Ramón y Cajal, Madrid, Spain; 6 Internal Medicine Department, Hospital Universitario Fundación Alcorcón, Madrid, Spain

**Keywords:** Hereditary hemorrhagic telangiectasia, Rare diseases, Angiogenesis, Coronavirus disease 2019 (COVID-19), Severe Acute Respiratory Syndrome Coronavirus-2 (SARS-CoV-2)

## Abstract

Hereditary hemorrhagic telangiectasia (HHT) is a rare autosomal-dominant disease characterized by pathologic angiogenesis that provokes vascular overgrowth. The evidence about the influence of Acute Respiratory Syndrome Coronavirus-2 (SARS-CoV-2) in patients with rare diseases is scarce. We aimed to know the prevalence of coronavirus disease 2019 (COVID-19) in HHT patients. The HHT pathogenic angiogenesis and endothelial injury in COVID-19 are discussed using data from RiHHTa (Computerized Registry of Hereditary Hemorrhagic Telangiectasia) registry. RiHHTa is an open, multicenter, prospective, observational registry including adult patients with HHT. A 27-item survey that captured clinical data of admitted HHT patients for COVID-19 was distributed to all RiHHTa investigators from June 8th to June 24th 2020. Only one out of 1177 HHT patients was admitted for COVID-19 pneumonia. She is a 74 years-old woman with a pathogenic variant in *ACVRL1* gene. Her clinical course did not involve mechanical ventilation or worsening epistaxis, and she was successfully discharged after two weeks. The endothelial damage and the consequent angiogenic process in COVID-19 patients deserve further investigation.

Dear Editor:

Hereditary hemorrhagic telangiectasia (HHT) is a rare autosomal-dominant disease characterized by vascular malformations. Disease-causing variants are mostly detected in endoglin (*ENG*; encoding endoglin) and activin A receptor type II-like 1 (*ACVRL1*; encoding activin receptor-like-kinase-1) genes. Both encoded proteins highly impact in angiogenesis as their loss-of-function provokes vascular overgrowth, mainly due to over-activation of phosphatidylinositol 3-kinase signalling [1].

The Severe Acute Respiratory Syndrome Coronavirus-2 (SARS-CoV-2) cause the coronavirus disease 2019 (COVID-19), that has spread worldwide [2, 3]. Since the COVID-19 outbreak in Wuhan (China), many articles addressing COVID-19 have been published. However, little is known about the influence of SARS-CoV-2 in patients with rare diseases, so there is a need for further clinical, translational and multicentric studies. Though knowledge of the pathogenic mechanisms of COVID-19 is still evolving, endothelial dysfunction can be considered a linchpin [4]. As previous data were lacking, we aimed to know the prevalence of COVID-19 pneumonia in HHT patients.

RiHHTa (Computerized Registry of Hereditary Hemorrhagic Telangiectasia) is an open, multicenter, prospective, observational registry including adult patients with HHT and developed within the Rare Diseases Working Group of the Spanish Society of Internal Medicine [1]. RiHHTa has an online design (accessible from https://rihhta.healthincode.com) available in Spanish or English with individual encoded access for each researcher and counted on the nonremunerated collaboration of the genetic studies company Health in Code (A Coruña, Spain). The design of the RiHHTa registry was approved by the Ethics Committee of the Hospital Universitari de Bellvitge. The rationale and methodology of RiHHTa have been published elsewhere [1]. For this study, we focused in those HHT patients who required admission for COVID-19 pneumonia, so we created a 27-item survey that captured clinical data of hospitalized patients with both diseases. From June 8th to June 24th 2020, an electronic survey was distributed to all RiHHTa investigators. The survey was responded by investigators from 22 (79.3%) out of the 29 Spanish hospitals collaborating with RiHHTa registry and include 1177 HHT patients followed-up by the RiHHTa investigators. Overall, only one patient was admitted for COVID-19 pneumonia. She is a 74 years-old woman with a pathogenic variant in *ACVRL1* gene and hepatic vascular involvement. Her clinical course did not involve mechanical ventilation or worsening epistaxis, and she was successfully discharged after 2 weeks.

This low prevalence of COVID-19 pneumonia among HHT patients may be due to several reasons. First, HHT patients could have self-isolated more strictly than non-HHT patients due to fear of COVID-19 therapeutic measures that might have worsened their recurrent epistaxis. Another possibility could be related to HHT pathogenesis. Observational data on lymphocytic endotheliitis suggest that SARS-CoV-2 provokes endothelial injury [2, 4]. A recent study compared morphologic and molecular features of lungs from COVID-19 patients’ autopsy with those who died from influenza and with uninfected controls. The lungs from COVID-19 patients had significant new vessel growth through conventional sprouting and an unexpected mechanism of intussusceptive angiogenesis [2]. In fact, recent evidence has shown higher Vascular Endothelial Growth Factor (VEGF) levels in COVID-19 patients compared with healthy controls [3]. Thus, bevacizumab, a monoclonal antibody against VEGF, is used in an ongoing single-group trial (NCT04275414) in critical COVID-19 patients. Moreover, in a recent study including 99 hospitalized COVID-19 patients, those in the intensive care unit (ICU) had significantly higher circulating endothelial cells counts than non-ICU patients and were correlated with inflammatory cytokines levels [5]. These findings provide in vivo evidence that the extent of endothelial injury is correlated with disease severity and that endothelial injury is a key feature of COVID-19. Whether or not the HHT pathogenic angiogenesis hinders endothelial damage in COVID-19 deserves further investigation.

## Data Availability

The datasets used and/or analysed during the current study are available from the corresponding author on reasonable request.

## Abbreviations

HHT: Hereditary hemorrhagic telangiectasia
ENG: Endoglin
ACVRL1: Activin A receptor type II-like 1
SARS-CoV-2: Severe Acute Respiratory Syndrome Coronavirus-2
COVID-19: Coronavirus disease 2019
RiHHTa: Computerized Registry of Hereditary Hemorrhagic Telangiectasia
VEGF: Vascular endothelial growth factor
ICU: Intensive care unit
